# Prevalence and risk factors of preoperative deep venous thrombosis in closed patella fracture: a prospective cohort study

**DOI:** 10.1186/s13018-021-02558-4

**Published:** 2021-06-23

**Authors:** Zhanchao Tan, Hongzhi Hu, Zhongzheng Wang, Yuchuan Wang, Yingze Zhang

**Affiliations:** 1 Department of Orthopaedic Surgery, The 3rd Hospital of Hebei Medical University, NO.139 Ziqiang Road, Shijiazhuang, 050051 Hebei China; 2 Department of Orthopedics, Union Hospital of Tongji Medical College of Huazhong University of Science and Technology, Wuhan, 430022 China; 3 Key Laboratory of Biomechanics of Hebei Province, Shijiazhuang, 050051 Hebei People’s Republic of China; 4 NHC Key Laboratory of Intelligent Orthopaedic Equipment, The Third Hospital of Hebei Medical University, Shijiazhuang, China

**Keywords:** Preoperative, DVT, Patella fracture, Risk factor

## Abstract

**Background:**

The preoperative prevalence of deep venous thromboembolism (DVT) of patella fracture is not well established. The study aimed to investigate the preoperative prevalence, the associated risk factors, and the locations of deep venous thrombosis (DVT) in patients with closed patella fracture.

**Methods:**

Patients who sustained closed patella fracture between January 1, 2016, and April 1, 2019, were included. Blood analyses and ultrasonography of bilateral lower extremities were routinely performed. Data of demographics, comorbidities, mechanism of injury, fracture type, total hospital stay, time from injury to DVT, and laboratory indexes were prospectively collected and compared between groups with and with non-DVT. Multivariate logistic regression analyses were performed to determine the independent risk factors of DVT.

**Results:**

Among the study cohort of 790 patients, 35 cases occurred in preoperative DVTs, indicating a prevalence of 4.4%, with 3.2% distal and 1.2% proximal DVT. Age ≥ 65 years old (OR, 3.0, 95% CI, 1.1–8.1), D-dimer > 0.5 mg/L (OR, 2.3, 95% CI, 1.1–4.8), and albumin < 35 g/L (OR, 2.5, 95% CI, 1.2–5.3) were identified to be risk factors of DVT in closed patella fracture. Among the DVTs, 30 cases (85.7%) occurred in the injured extremity, 3 cases (8.6%) in bilateral extremities, and 2 cases (5.7%) solely in the uninjured extremity.

**Conclusion:**

The prevalence of preoperative DVT in closed patella fracture was 4.4%, with 3.2% for distal and 1.2% for proximal DVT. We recommend individualized risk stratification and early anticoagulation for patients with risk factors (age ≥ 65 years, D-dimer > 0.5 mg/L and albumin < 35g/L).

## Background

Patella fracture, a kind of intraarticular fracture, was reported to account for approximately 1–2.6% of fractures in adults, and the elderly above 60 years is a high incidence of the population, and there was an increasing trend with ages [1–3]. Most patella fractures need surgical treatment and a satisfactory outcome can be obtained through surgical treatment and postoperative rehabilitation [4]. DVT (deep vein thrombosis) is a dangerous complication in orthopedic patients that can lead to adverse clinical outcomes, such as life-threatening pulmonary embolism (PE). Preoperative DVT may cause a surgical delay in orthopedic patients, which would significantly increase postoperative morbidity and mortality and have an adverse effect on the quality of life [5–8]. Ignoring the progression of DVT in such a population often leads to a bad prognosis, while an early simple intervention often obtains a satisfactory outcome.

Generally, plasma hypercoagulability after trauma, venous stasis, and injury of venous endothelium are believed to be predisposing factors of DVT [9]. In the specific clinical context, such as patella fracture in geriatric patients, immobilization of the knee and surgical delay due to comorbidities may predispose patients to DVT. DVTs in patients who underwent major orthopedic surgeries (total joint arthroplasty and hip and pelvic fracture surgery) have been extensively studied [8, 10, 11]. However, the detailed information on the prevalence, related risk factors, and locations of DVT in isolated lower limb fractures (ILLFs) has not been paid much attention to. There were few kinds of researches focusing on the DVT in ILLFs of different anatomical sites [12–15], and the incidence rates varied greatly between different locations. To our knowledge, the current study was the first study with an adequate size sample focusing on the preoperative DVT in closed patella fractures.

It is of great clinical significance to identify the risk factors of DVT and determine the optimal thromboprophylaxis. Although the predominant DVT in ILLFs is distal DVT [13–15], it has been reported that trauma-induced distal DVT can propagate proximally [16], significantly increasing the risk of life-threatening PE. Currently, controversy still exists on thromboprophylaxis in patients with ILLFs. Even the clinical guidelines of different countries are not consistent on this clinical issue [17, 18]. In summary, the understanding of preoperative DVT in closed patella fractures is currently limited. Risk stratification of preoperative DVT in such a population has not been studied. Thus, the primary purpose of the study was to investigate the incidence and risk factors of preoperative DVT in closed isolated patella fractures and the secondary purpose was to investigate the locations of the DVT.

## Patients and methods

This was a prospective study. The methods of the study were conducted in accordance with the STROCSS (Strengthening the Reporting of Cohort Studies in Surgery) guidelines. Approval from the ethics committee of our institution was acquired. All the participants had written informed consent. Patients who sustained acute closed patella fracture between January 1, 2016, and April 1, 2019, in the level-I trauma center were included. Blood analyses and ultrasonography of bilateral lower extremities were routinely performed. According to the empirical criteria, the sample size of logistic regression analysis should be 10 to 15 times the number of covariates. There were 33 covariates in this study, and the sample size should not be less than 33 × 10 = 330.

### Inclusion and exclusion

Patients who met the following criteria were included: 1. age > 18 years; 2. diagnosis of closed isolated patella fracture; 3. with complete and available medical data. Patients with pathological fractures, old fractures (treatment delayed > 3weeks), and polytrauma were excluded. Besides, patients who were under anticoagulant treatment on admission for other illnesses were also excluded.

**Fig. 1:**
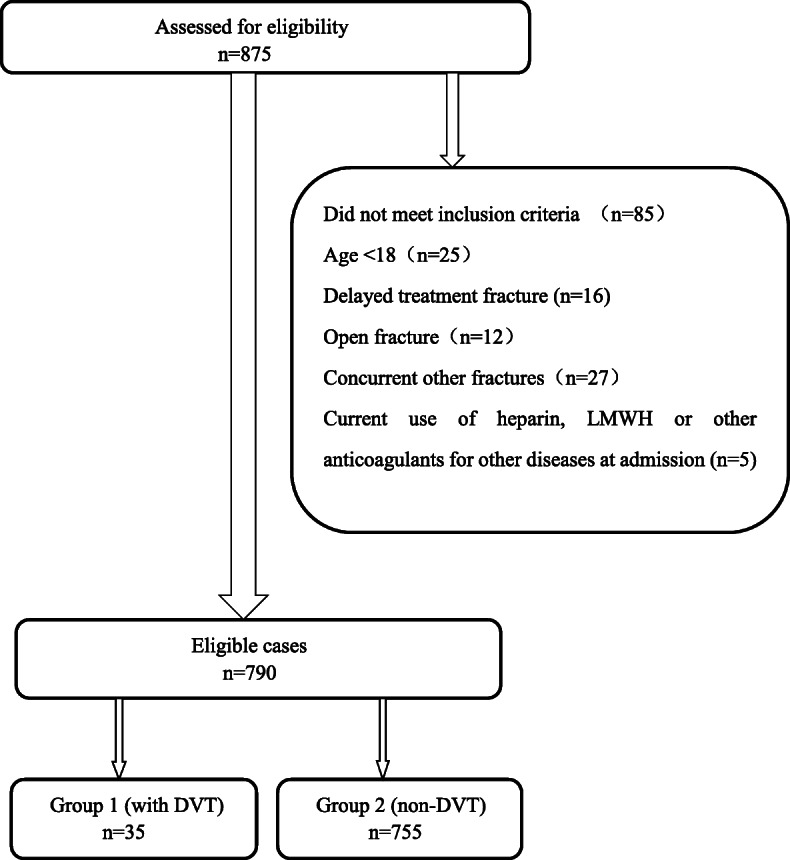
Exclusion criteria and the eligible cases included in this study

A total of 875 patients with unilateral patella fracture were admitted to our institution for surgery treatment between January 1, 2016, and April 1, 2019. Among these patients, 790 cases met the criteria and agreed to participate in the study, consisting of 469 males and 321 females, with a median age of 53 (interquartile range (IQR), 18 to 92). The participants were divided into two groups based on the occurrence of preoperative DVT: group 1 for DVT; group 2 for non-DVT (Fig. 1).

After admission, the injured limbs of the patients received splinting and were elevated appropriately. All the participants received pharmacotherapy (low molecular weight heparin, 2500–4100 IU once daily, subcutaneous injection).

### Diagnosis of DVT

Duplex ultrasonography (DUS) was used to diagnose DVT. All the participants went through DUS of bilateral lower extremities at admission, subsequently every 3 days and any time when a DVT was suspected. The diagnostic criteria for DVT were non-compressed vein, lumen obstruction or filling defect, the lack of respiratory vibration above the knee vein segment, and inadequate flow augmentation to the calf [19]. Duplex ultrasound scanning included the common femoral vein, superficial femoral and deep femoral vein, popliteal vein, anterior tibial vein, posterior tibial vein, and common fibular vein. Blood clot located in the intermuscular vein (gastrocnemius veins and soleal veins) was excluded in this study. DVT that occurred in the popliteal vein and above was defined as proximal DVT. DVT that occurred below the popliteal vein was defined as distal DVT.

### Data collection

The data we used in the study was from a database, which was set up to prospectively investigate the complications in patients with different fractures in the institution. The detailed medical data was prospectively collected using a standard chart.

The data included demographic variables, comorbidities, injury mechanism (low or high energy), fracture types (simple or comminuted), history of cerebral infarction, history of any surgery, total hospital stay, time from injury to DVT (time from injury to the initial diagnosis of DVT), and laboratory indexes at admission. Demographic variables included age, gender, body mass index (BMI), current smoking, alcohol consumption, and living place (urban or rural); comorbidities included hypertension, diabetes mellitus, ischemic heart disease, arrhythmia, and chronic lung diseases. Fall while walking was defined as low-energy injury; other damages were regarded as high-energy injury. BMI was categorized into four groups: underweight (< 18.5), normal (18.5–23.9), overweight (24.0–27.9), and obesity (≥ 28.0). Age was grouped into three categories: 18–44 years old, 45–64 years old, and above 65 years old. The laboratory indexes at admission included complete blood count and biochemical analysis.

### Statistical analysis

Continuous variables were analyzed by Student’s t test for independent samples or Mann-Whitney U test. Categorical variables were analyzed by chi-square or Fisher’s exact test. Multivariate logistic regression analysis was used to identify the risk factors. OR (odds ratio) and 95% CI (confidence interval) were used to express correlation strength. *P* < 0.05 was set to be the statistical level. Hosmer-Lemeshow (H-L) test was performed to assess the fitting degree of the final model. The statistical software used in the study was SPSS22.0 (IBM, Armonk, New York, USA).

## Results

Among the study cohort of 790 patients, 35 cases developed a preoperative DVT, indicating a prevalence of 4.4%, with 25 cases (3.2%) distal and 10 cases (1.2%) proximal DVT. Among the DVTs, 30 cases (85.7%) occurred in the injured extremity, 3 cases (8.6%) in bilateral extremities, and 2 cases (5.7%) solely in the uninjured extremity. At the first Doppler ultrasound scanning after admission, no DVTs were found in the uninjured limbs. Compared with patients with non-DVT, patients with DVT had a longer total hospital stay (8.7 ± 2.1 vs 13.9 ± 1.3, *P* < 0.05). The comparison results of variables between group 1 and group 2 were presented in Table 1. Significant differences were found in seven variables, including age category, injury mechanism (high energy), alcohol consumption, D-dimer > 0.5 mg/L, albumin < 35 g/L, platelet (PLT) > 300 ×10^9^/L, and low-density lipoprotein cholesterol (LDL-C) > 3.37 mmol/L, (*p* < 0.05). The seven variables were entered into the final model of the multivariate analysis and three variables were identified to be independent risk factors of the preoperative DVT in closed isolated patella fracture, including age ≥ 65years old, (OR, 3.0, 95% CI, 1.1–8.1), D-dimer > 0.5 mg/L, (OR, 2.3, 95% CI, 1.1–4.8), and albumin < 35 g/L, (OR, 2.5, 95% CI, 1.2–5.3), (Table 2). H-L Test revealed well fitness of the model (X^2^ = 6.768, *p* = 0.831).

**Table 1:** Comparison between group 1 and group 2 in all variables collected

Variables	Group 1 (*n* = 755)	Group 2 (*n* = 35)	p
	Number (%)	Number (%)	
Gender(male)	445 (58.9)	24 (68.6)	.257
Age category			.003
18< Age ≤ 44 years	242 (32.1)	8 (22.9)	
45 ≤ Age < 64 years	367 (48.6)	12 (34.3)	
Age ≥ 65 years	146 (19.3)	15 (42.9)	
BMI (kg/m^2^)			.973
18.5–23.9	455 (60.3)	23 (65.7)	
< 18.5	25 (3.3)	1 (2.9)	
24.0–27.9	202 (26.8)	8 (22.9)	
≥ 28.0	73 (9.7)	3 (8.6)	
Current smoking	99 (13.1)	5 (14.3)	.799
Alcohol consumption	68 (9.0)	7 (20.0)	.030
Living place (rural)	392 (51.9)	20 (57.1)	.545
Diabetes mellitus	81 (10.7)	4 (11.4)	.783
Hypertension	167 (22.1)	8 (22.9)	.918
Ischemic heart disease	52 (6.9)	2 (5.7)	.566
Arrhythmia	34 (4.5)	3 (8.6)	.222
Chronic lung diseases	11 (1.5)	1 (2.9)	.422
History of cerebral infarction	34 (4.5)	3 (8.6)	.222
History of any surgery	130 (17.2)	4 (11.4)	.372
Injury mechanism (high energy)	91 (12.1)	10 (28.6)	.009
Fracture type (comminuted)	78 (10.3)	6 (17.1)	.253
TP (< 60 g/L)	360 (47.7)	20 (57.1)	.273
ALB (< 35 g/L)	221 (29.3)	21 (60.0)	.000
FBG (> 6.1 mmol/L)	228 (30.2)	9 (25.7)	.571
TC (> 5.2 mmol/L)	63 (8.3)	3 (8.6)	.962
TG (> 1.7 mmol/L)	160 (21.2)	6 (17.1)	.565
LDL-C (> 3.37 mmol/L)	205 (27.2)	15 (42.9)	.043
VLDL (> 0.78 mmol/L)	158 (20.9)	6 (17.1)	.589
HDL-C (< 1.1 mmol/L)	206 (27.3)	11 (31.4)	.591
WBC (> 10 × 10^9^/L)	152 (20.1)	6 (17.1)	.666
NEUT (> 6.3 × 10^9^/L)	268 (35.5)	8 (22.9)	.125
LYM (< 1.1 × 10^9^/L)	121 (16.0)	6 (17.1)	.860
RBC (< lower limit)	167 (22.1)	7 (20.0)	.767
HGB (< lower limit)	174 (23.0)	6 (17.1)	.416
HCT (< lower limit)	141 (18.7)	6 (17.1)	.820
PLT (> 300 × 10^9^/L)	47 (6.2)	6 (17.1)	.024
RDW (> 16.5%)	7 (0.9)	1 (2.9)	.305
PDW (> 18.1%)	16 (2.1)	1 (2.9)	.541
D-dimer (> 0.5 mg/L)	352 (46.6)	27 (77.1)	.000

*DVT* deep venous thrombosis, *BMI* body mass index, *RBC* red blood cell, reference range: female, 3.5–5.0 × 10^12^/L, males, 4.0–5.5 × 10^12^/L, *HGB* hemoglobin, reference range: females, 110–150 g/L, males, 120–160 g/L, *FBG* fasting blood glucose, *HCT* hematocrit, 40–50%, *WBC* white blood cell, *NEUT* neutrophile, *LYM* lymphocyte, *PLT* platelet, 100–300 × 10^9^/L, *TP* total protein, *ALB* albumin, *RDW* red cell distribution width, *PDW* platelet distribution width, *TC* total cholesterol, TG triglyceride, *LDL-C* low-density lipoprotein cholesterol, *HDL-C* high-density lipoprotein cholesterol, *VLDL* very-low-density lipoprotein

**Table 2:** Multivariate analyses of risk factors associated with preoperative DVT in closed isolated patella fracture

Risk factors	OR (95% CI)	*P* value
D-dimer (> 0.5 mg/L)	2.3 (1.1–4.8)	.032
Albumin (<35g/L)	2.5 (1.2–5.3)	.017
Age category		.045
18< Age ≤ 44 years	Reference	
45 ≤ Age < 64 years		.624
Age ≥ 65 years old	3.0 (1.1-8.1)	.028

*DVT* deep venous thrombosis, *OR* odds ratio, *CI* confidence interval

## Discussion

The current study firstly investigated the preoperative DVT in closed patella fracture. Based on the results of the study, we found that the prevalence of preoperative DVT in closed patella fracture was 4.4%, with 3.2% distal and 1.2% proximal DVT. Three variables, including age > 65 years, D-dimer > 0.5 mg/L, and albumin < 35 g/L were identified to be independent risk factors of DVT in closed patella fracture.

Studies focusing on the incidence of DVT in patella fractures were currently limited; only two studies investigated the prevalence of DVT in patella fractures; neither evaluated the potential risk factors of DVT. Jared et al. [15] conducted a retrospective study to estimate the annual incidence of thrombotic events in 30 days in different lower limb fractures from 2008 to 2018; he found the overall incidence of DVT in patella fracture was 0.6%. In his study, all the DVTs were symptomatic, while asymptomatic DVTs were not included, which may account for the lower incidence of DVT in his study. Wang et al. [14] conducted a research to investigate the perioperative DVT rate in specific isolated lower extremity fractures, in which a small sample of 59 patients with patella fracture was included. The overall preoperative DVT rate in patella fracture was 15.3% (9/59), including proximal DVT (1.7%, 1/59) and distal DVT (13.6%, 8/59), which is much higher than that of the current study. Blood clots located in the intermuscular veins were excluded in the current study, while included in the study of Wang et al. [14], which may account for the wide difference of the DVT rate between the two researches.

In the current study, 85.7% of the DVTs occurred in the injured legs, 8.6% in bilateral legs, and 5.7% solely in the uninjured extremity. The results indicated that DVT in closed isolated patella fracture could occur in both injured and uninjured legs and there is a tendency of higher incidence in the injured legs. The finding was similar to some previous researches [20]. Wang et al. [14] reported that 83.6% of the DVTs were located in the injured extremity, 5.4% in the bilateral extremity, and 11.0% solely in the uninjured extremity in patients with isolated lower limb fracture. Niu et al. [21] found that 76.1% of the DVTs solely involved the injured leg, 9.0% involved bilateral legs, and 14.9% solely involved the uninjured legs in the geriatric patients with a femoral neck fracture. However, some studies got slightly different results. Shuang et al. [22] reported that in patients with lower extremity fractures, 60% of the preoperative DVTs were located in bilateral legs and 40% were solely located in the uninjured legs. Immobilization of the injured legs and vascular endothelial injury caused by fractures may account for the predominant rate of DVT that occurred in the injured legs.

We also found that the majority of the DVTs in closed patella fracture were distal DVT. In contrast with this, the rate of proximal DVT in the hip was much higher [8, 11, 21, 23]. This demonstrated that the location of DVT may be associated with the fracture anatomical sites, the more proximal to the hip of the fracture site, the higher risk of the proximal DVT.

Old age has been well demonstrated to be a risk factor of DVT among orthopedic patients by previous studies [24]. Park et al. [25] reported that an advanced age above 60 years was a significant predictor of DVT in patients with fractures below the hip. In a prospective cohort study, Zhang et al. [26] reported that age over 65 years was associated with the formation of preoperative DVT in distal femur fractures. Goel et al. [27] found age > 40 years was an independent predictor for DVT following fractures below the knee. Zhang et al. [28] conducted a retrospective study and found that age was a significant risk factor of preoperative DVT in hip fractures. Consistent with these studies, in the current study, we found age ≥ 65 years was a significant risk factor of preoperative DVT in closed patella fracture. We recommend routine anticoagulation and preoperative DVT screening in elderly patients above 65 years with patellar fractures.

D-dimer, a specific degradation product derived from fibrinolytic cross-linked fibrin clots, has been widely applied as a predictor of DVT in patients. The diagnosis value of D-dimer in thrombotic events has been well established [29, 30]. Though D-dimer indicates higher sensitivity, the specificity is low. Some researchers demonstrated that the combination of age and D-dimer as a critical value significantly improved the accuracy of prediction for the occurrence of DVT [31, 32]. Reagh et al. [33] found that D-dimer levels had differences between males and females; however, there was no significant difference in optimal cutoff value for excluding DVT between genders. The current study confirmed that an elevated level of D-dimer (> 0.5 mg/L) was correlated with an increased risk of DVT in closed patella fractures.

Serum albumin level is an indicator of nutritional status. Albumin less than 35 g/L is defined as hypoalbuminemia [34]. Serum albumin level plays an important role in patients’ prognosis in many medical contexts. Preoperative albumin level was an important indicator for preoperative evaluation of complications and prognosis in orthopedic patients [35]. Hypoalbuminaemia was reported to be an important trigger factor in the elevation of fibrinogen and platelet aggregability, which can be reversed by infusing albumin [36, 37]. In a prospective study, Ma et al [12] found that albumin < 35 g/L was an independent risk factor of DVT in lower limb fracture. Lung et al. [38] reported lower serum albumin was significantly correlated with an increased rate of DVT in patients who underwent total shoulder arthroplasty. Consistent with these studies, in the current study, we found that low albumin < 35 g/L was an independent risk factor of DVT in closed patella fracture. Hypoalbuminemia was a modifiable risk factor of DVT in such a population; we recommend preoperative screening and correction of nutritional status in patients with patella fracture, though the optimal strategy for correcting serum albumin levels to reduce the risk of DVT remains uncertain and further studies are needed.

Some inherent limitations existed in the current study. First, this was a single-center study; there may be a selection bias of the study cohort, and further multicenter studies are necessary. Second, like other multivariate analyses, we could not include every potential risk factor in the study and some potential risk factors might be missed. Third, data on the variable of comorbidity were collected based on the self-report of the patient, and this may influence the accuracy of the data. Fourth, the participants in the study were all inpatients for surgical treatment; those outpatients were not involved; thus, there may be a selection bias to some extent.

## Conclusion

The prevalence of preoperative DVT in closed patella fracture was 4.4%, with 3.2% for distal and 1.2% for proximal DVT. We recommend individualized risk stratification and early anticoagulation for patients with high-risk factors (age ≥ 65 years, D-dimer > 0.5mg/L, albumin < 35g/L).

## Data Availability

All the data will be available upon motivated request to the corresponding author of the present paper.
